# Post-dural Puncture Headache: Pathophysiology, Risk Stratification, Prevention, and Evidence-Based Management for Practicing Anesthesiologists

**DOI:** 10.7759/cureus.106499

**Published:** 2026-04-06

**Authors:** Rayees A Konduru, Arshiya Shabnam, Joel Yarmush, Hattiangadi Sangeetha Kamath

**Affiliations:** 1 Anesthesiology, NewYork-Presbyterian Brooklyn Methodist Hospital, Brooklyn, USA; 2 Internal Medicine, New York Medical College, Valhalla, USA

**Keywords:** epidural blood patch, headache disorders, intracranial hypotension, lumbar puncture, neuraxial anesthesia, obstetric anesthesia, post-dural puncture headache

## Abstract

Post-dural puncture headache (PDPH) is a well-recognized secondary headache disorder resulting from persistent cerebrospinal fluid (CSF) leakage following iatrogenic dural disruption during neuraxial procedures, including spinal anesthesia, diagnostic lumbar puncture, dural puncture epidural (DPE), and unintentional dural puncture (UDP). The syndrome reflects a complex interaction between CSF hydrodynamics, craniospinal compliance, vascular compensation, and nociceptive sensitization. Reduction in CSF volume produces intracranial hypotension and compensatory venous dilation in accordance with the Monro-Kellie doctrine, while loss of CSF buoyancy permits caudal displacement of intracranial structures, resulting in traction on pain-sensitive meninges and cranial nerves and generating the characteristic orthostatic headache. Procedural variables, particularly needle gauge and tip geometry, represent the most important modifiable determinants of PDPH risk. Incidence varies from less than 3% following spinal anesthesia with small-gauge atraumatic needles to 50-85% after UDP with large-bore Tuohy needles, whereas DPE techniques demonstrate comparatively low incidence (approximately 0.5-2%). Management ranges from conservative therapy with hydration, analgesics, and caffeine to targeted pharmacologic therapies and epidural blood patch, which remains the gold-standard intervention with success rates approaching 70-90%. Emerging evidence implicating glymphatic dysfunction, neuroinflammatory mechanisms, and advanced imaging-based leak localization may facilitate improved risk stratification and individualized therapeutic strategies.

## Introduction and background

Post-dural puncture headache (PDPH) is a well-recognized secondary headache disorder resulting from disruption of the meningeal barrier following dural puncture [1]. This complication most commonly occurs after spinal anesthesia, epidural anesthesia complicated by unintentional dural puncture (UDP), or diagnostic lumbar puncture performed for neurologic evaluation. Although PDPH is frequently self-limited, it remains one of the most clinically significant complications associated with neuraxial procedures and continues to represent an important source of morbidity in both anesthetic and neurologic practice.

The syndrome was first systematically characterized in the mid-20th century by Vandam and Dripps, whose longitudinal observations provided foundational insights into the epidemiology, natural history, and risk factors associated with PDPH [2]. Their work established PDPH as a reproducible clinical entity rather than a nonspecific postoperative complaint and highlighted procedural variables, particularly needle gauge and tip design, as critical determinants of risk.

Contemporary epidemiologic studies have further refined the understanding of PDPH incidence across different neuraxial techniques. Following a diagnostic lumbar puncture performed with cutting needles, the incidence of PDPH ranges from 10-40% [3], whereas the use of atraumatic pencil-point needles reduces the incidence to less than 3%. Similarly, spinal anesthesia performed with small-gauge (25-27 G) atraumatic needles is associated with an incidence generally below 3% [4].

The incidence of PDPH following the dural puncture epidural (DPE) technique appears to be comparatively low, typically ranging from approximately 0.5-2% when fine-gauge atraumatic spinal needles are used [5]. This reduced risk likely reflects the small dural defect produced by pencil-point needles combined with limited cerebrospinal fluid (CSF) leakage. In contrast, UDP during epidural placement is associated with a markedly higher incidence of PDPH, ranging from 50-85%, due to the large diameter of Tuohy needles and sustained CSF leakage [6].

Large observational analyses, including multicenter cohort studies, such as those by Kwak et al., consistently demonstrate that needle gauge and tip configuration remain the strongest procedural predictors of PDPH risk [7]. Patient-related factors, including younger age, female sex, pregnancy, and lower body mass index, further increase susceptibility [8]. Obstetric patients represent a uniquely high-risk population due to physiologic venous engorgement, hormonal influences on connective tissue, and the high frequency of neuraxial analgesia utilization during labor.

The clinical consequences of PDPH extend beyond headache-related discomfort. In obstetric populations, PDPH may impair maternal-neonatal bonding, delay mobilization, increase reliance on opioid analgesics, prolong hospitalization, and contribute to increased healthcare resource utilization. In rare but serious cases, persistent intracranial hypotension resulting from continued CSF leakage may lead to complications such as subdural hematoma or cerebral venous sinus thrombosis.

Despite advances in needle design and technique that have substantially reduced incidence, PDPH remains clinically relevant due to its multifactorial pathophysiology, variable presentation, and potential for significant morbidity [1]. A comprehensive understanding of the mechanisms, risk factors, and management strategies associated with PDPH is therefore essential to optimize patient outcomes and guide evidence-based neuraxial practice.

## Review

Clinical presentation

PDPH most commonly presents as a bilateral frontal, occipital, or generalized headache that exhibits a characteristic orthostatic pattern [1]. Patients typically report worsening symptoms within minutes of assuming an upright posture, with substantial improvement when lying supine. This positional component remains one of the most distinctive clinical features of the disorder.

The headache may be described as dull, throbbing, or pressure-like in quality and is frequently accompanied by a constellation of associated symptoms. These may include cervical stiffness, nausea, vomiting, photophobia, phonophobia, tinnitus, hearing disturbances, dizziness, and visual symptoms [1]. Diplopia resulting from sixth cranial nerve palsy is among the most commonly described neurologic manifestations and reflects traction on the abducens nerve due to intracranial hypotension [9].

The onset of symptoms typically occurs within 48 to 72 hours following dural puncture, although earlier and delayed presentations have been described [1].

Diagnostic criteria and differential diagnosis

The International Classification of Headache Disorders defines PDPH as a headache occurring within five days of dural puncture that is aggravated by upright posture and resolves spontaneously within two weeks or after treatment with an epidural blood patch (EBP) [10].

Although the majority of patients experience spontaneous resolution within five to seven days, a subset of individuals may develop prolonged or persistent headache lasting several weeks or months [11]. Recent observational studies in obstetric populations have suggested that patients who experience UDP may also demonstrate increased rates of postpartum headache and chronic pain syndromes extending beyond the immediate postpartum period.

The differential diagnosis of postpartum headache following neuraxial anesthesia is broad and includes both primary and secondary etiologies that may mimic PDPH. Important alternative diagnoses include primary headache disorders such as migraine and tension-type headache, as well as serious secondary conditions, including preeclampsia or eclampsia, cerebral venous sinus thrombosis, subdural hematoma, meningitis, reversible cerebral vasoconstriction syndrome, intracranial hemorrhage, and pituitary apoplexy [12].

A thorough clinical assessment, including blood pressure evaluation, neurologic examination, and laboratory testing, may be required to differentiate these conditions. Neuroimaging is not routinely necessary when the clinical presentation is typical; however, imaging studies are recommended when patients present with atypical features, focal neurologic deficits, seizures, or persistent symptoms despite appropriate therapy.

Magnetic resonance imaging (MRI) with gadolinium contrast [12] is considered the preferred modality, as it allows evaluation of intracranial hypotension and exclusion of complications such as subdural hematoma or venous sinus thrombosis. Routine repeat lumbar puncture for CSF pressure measurement is generally discouraged because it may exacerbate CSF leakage and worsen symptoms.

Early recognition of alternative diagnoses is essential to ensure timely and appropriate management.

Pathophysiologic mechanisms

**Figure 1:**
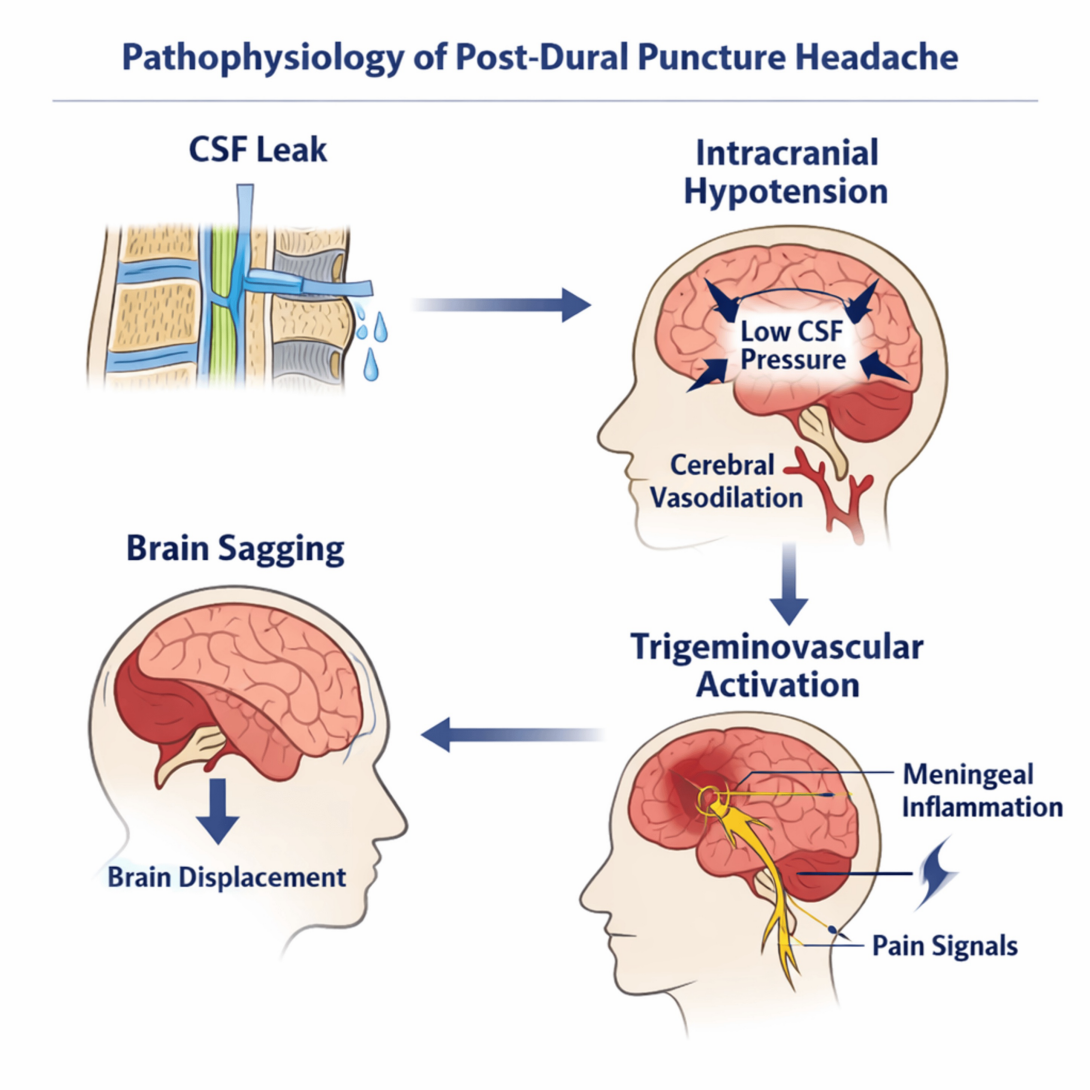
Proposed pathophysiologic mechanisms of post-dural puncture headache (PDPH) Following dural disruption, persistent CSF egress lowers intracranial CSF volume. In accordance with the Monro-Kellie doctrine, compensatory cerebral venous dilation occurs to maintain intracranial volume homeostasis. Reduced CSF buoyancy permits downward displacement of intracranial contents, producing traction on meninges and cranial nerves. These interacting mechanisms collectively generate the orthostatic pain phenotype characteristic of PDPH. Image credit: The authors. Figure created with BioRender.com.

The pathophysiology of PDPH is complex and multifactorial (Figure 1). Contemporary models incorporate mechanical, vascular, compliance-related, and inflammatory mechanisms. No single explanatory framework fully accounts for the variability in symptom severity, imaging findings, and treatment response observed among affected patients.

CSF Volume Depletion and the Monro-Kellie Doctrine

The classical explanation for PDPH centers on persistent CSF leakage through a dural defect, resulting in decreased intracranial CSF volume and intracranial hypotension. Mokri refined this concept by integrating it with the Monro-Kellie doctrine, which states that the combined volumes of brain tissue, CSF, and intracranial blood remain constant within the rigid cranial vault [13].

When CSF volume decreases due to leakage, compensatory expansion of the intracranial venous system occurs to maintain total intracranial volume. This compensatory venous dilation primarily affects dural sinuses and meningeal veins and may result in distension of pain-sensitive structures within the cranial vault, thereby generating headache.

Neuroimaging findings observed in patients with intracranial hypotension, including venous sinus enlargement, engorgement of meningeal veins, and diffuse pachymeningeal enhancement, support this vascular compensation model [14]. However, vascular dilation alone cannot fully explain the orthostatic nature of symptoms or the occurrence of cranial neuropathies, suggesting that additional mechanisms contribute to the clinical syndrome.

Caudal Brain Displacement and Neural Traction

Another widely accepted mechanism involves downward displacement of intracranial structures resulting from loss of CSF buoyancy. Under normal physiologic conditions, CSF provides buoyant support that reduces the effective weight of the brain within the cranial vault. When CSF volume decreases, this support is diminished, allowing gravitational forces to produce downward displacement of brain structures.

Rando and Fishman proposed that traction on pain-sensitive meninges and cranial nerves plays a central role in generating the orthostatic pain characteristic of PDPH [15]. MRI findings associated with this process may include cerebellar tonsillar descent, pituitary enlargement, effacement of basal cisterns, and the presence of subdural fluid collections.

Certain cranial nerves are particularly vulnerable to traction injury due to their long intracranial courses. The abducens nerve is especially susceptible, which explains the frequent occurrence of diplopia in patients with severe intracranial hypotension [9]. Vestibulocochlear symptoms, such as tinnitus and hearing changes, may similarly reflect traction at the cerebellopontine angle.

Interestingly, the severity of radiographic findings does not consistently correlate with symptom intensity. This observation suggests that other factors, including variations in craniospinal compliance or inflammatory sensitization, may influence clinical presentation.

Craniospinal Compliance and Leak Kinetics

Structural properties of the dura mater and surrounding spinal tissues play an important role in determining the persistence and severity of CSF leakage. Investigations in spontaneous intracranial hypotension suggest that connective tissue composition and dural elasticity influence the ability of dural defects to close spontaneously.

Procedural PDPH differs from spontaneous CSF leak syndromes in several important respects. Bezov and colleagues emphasized that the geometry of the dural defect varies depending on needle design [1]. Atraumatic pencil-point needles typically create slit-like defects that separate dural fibers without cutting them, allowing the tissue to reapproximate following needle removal. In contrast, cutting needles create circular defects that may permit sustained CSF leakage.

Histologic analyses by Reina et al. confirm that atraumatic needles preserve the architecture of dural fibers, whereas cutting needles transect these fibers, thereby increasing the likelihood of persistent leakage [16].

Variability in spinal epidural compliance and venous capacitance may also influence symptom severity. Differences in connective tissue properties among individuals may explain why some patients experience severe and prolonged symptoms while others demonstrate rapid recovery despite similar procedural factors.

Neuroinflammatory Contributions

Although PDPH has historically been considered a purely mechanical phenomenon, emerging evidence suggests that inflammatory mechanisms may contribute to symptom development. Experimental studies have demonstrated that meningeal irritation can activate sterile inflammatory pathways involving cytokine release and trigeminovascular sensitization [17].

These inflammatory processes may amplify nociceptive signaling and contribute to variations in pain severity among patients with comparable CSF leak characteristics. While direct human evidence remains limited, this emerging framework highlights the potential role of neuroinflammation in modulating PDPH symptomatology.

Future investigations exploring inflammatory biomarkers may help clarify this component of PDPH pathophysiology and potentially identify new therapeutic targets.

Glymphatic and CSF Hydrodynamic Considerations

Emerging research suggests that CSF hydrodynamics and glymphatic circulation may contribute to PDPH symptom generation. Experimental imaging studies have demonstrated that reductions in CSF pressure alter the transmantle pressure gradient and glymphatic flow, potentially influencing interstitial fluid clearance and nociceptive signaling pathways. Schievink and colleagues have suggested that alterations in spinal epidural venous pressure and dural compliance may determine whether a dural defect results in persistent CSF leakage versus rapid spontaneous closure [12]. These factors may explain the heterogeneity in symptom severity among patients with similar procedural characteristics [18].

Risk factors

Patient-Related Factors

Several patient characteristics have been associated with increased susceptibility to PDPH. Younger age remains one of the most consistently reported risk factors, potentially reflecting greater dural elasticity and higher baseline CSF pressure in younger individuals [8].

Female sex has also been associated with increased risk, although the underlying mechanisms remain uncertain. Hormonal influences and differences in connective tissue properties have been proposed as potential explanations [8].

A prior history of primary headache disorders, including migraine or tension-type headache, may further increase susceptibility to PDPH [1]. Pregnancy itself represents an independent risk factor due to physiologic changes such as increased venous engorgement and hormonal modulation of connective tissue structures.

Evidence regarding the relationship between body mass index and PDPH risk remains inconsistent, with some studies suggesting protective effects of higher BMI while others demonstrate no significant association.

Procedural Factors

Procedural variables play a critical role in determining the risk of PDPH, with needle design representing the most important modifiable factor (Table 1). Numerous studies have demonstrated that atraumatic pencil-point needles significantly reduce PDPH incidence compared with traditional cutting needles [4]. Cutting needles, such as Quincke tips, create sharp circular dural defects that tend to remain patent, whereas atraumatic pencil-point needles (e.g., Whitacre, Sprotte) separate rather than transect dural fibers, facilitating tissue reapproximation after needle removal and promoting spontaneous sealing of the dural defect [16]. Histologic investigations by Reina et al. support this mechanism, demonstrating preservation of dural fiber integrity with pencil-point needles compared with clear fiber transection produced by cutting needles [16].

**Table 1:** Incidence of post-dural puncture headache (PDPH)

Procedure	Needle type	Approximate PDPH incidence
Diagnostic LP (Quincke 20-22 G)	Cutting	10-36%
Diagnostic LP (25-27 G Whitacre/Sprotte)	Atraumatic (pencil-point)	0.5-3%
Spinal anesthesia (25-27 G pencil-point)	Atraumatic	0.5-2%
Dural puncture epidural (DPE) technique	Fine atraumatic spinal needle	0.5-2%
Unintentional dural puncture (Tuohy 16-18 G)	Large cutting epidural needle	50-80%

Needle gauge also significantly influences PDPH risk, with larger diameter needles associated with a higher incidence. Reported PDPH rates range from approximately 15-36% with 22-gauge Quincke needles and 3-15% with 25-gauge Quincke needles. In contrast, pencil-point needles substantially reduce risk, with incidences of approximately 1-3% reported for 25-gauge Whitacre or Sprotte needles and less than 1-2% for 27-gauge atraumatic needles. The risk is markedly higher following UDP with large epidural Tuohy needles (16-18 gauge), where PDPH incidence ranges from 50 to 85%, reflecting the larger dural defect created by these needles.

Multiple systematic reviews confirm the strong influence of needle characteristics on PDPH incidence. A meta-analysis by Halpern and Preston involving more than 4,500 spinal anesthetics demonstrated that non-cutting pencil-point needles reduce PDPH incidence by nearly 70% compared with Quincke needles [19]. Similarly, the Cochrane review by Arevalo-Rodriguez et al. concluded that smaller-gauge atraumatic needles significantly decrease PDPH risk without compromising procedural success rates [4]. Although very fine needles may increase technical difficulty, procedure time, and failure rates, most evidence supports the use of small-gauge atraumatic needles as the optimal balance between procedural success and minimization of PDPH risk.

Additional procedural factors associated with increased PDPH risk include multiple dural puncture attempts, bevel orientation perpendicular to longitudinal dural fibers, and operator inexperience [7]. In obstetric anesthesia, the relatively large diameter of epidural Tuohy needles contributes to the substantially higher incidence of PDPH following UDP compared with spinal anesthesia performed using fine-gauge atraumatic needles.

Preventive strategies

Multiple preventive strategies have been investigated to reduce the incidence of PDPH following dural puncture.

Strict bed rest and aggressive hydration were historically recommended; however, systematic reviews and Cochrane analyses have demonstrated that these interventions do not significantly reduce PDPH incidence [20].

Among procedural interventions, atraumatic needle design remains the most effective preventive measure [4]. The Cochrane systematic review by Arevalo-Rodriguez et al. confirmed that pencil-point needles significantly reduce PDPH incidence compared with cutting needles across a variety of clinical settings. Other proposed strategies include bevel orientation parallel to dural fibers during lumbar puncture, minimizing the number of puncture attempts, and utilizing ultrasound guidance in technically challenging patients [4]. 

Caffeine has been explored as a prophylactic therapy due to its vasoconstrictive effects on cerebral vessels. Although caffeine may provide temporary symptomatic improvement, evidence supporting routine prophylactic use remains limited. 

Cosyntropin, a synthetic adrenocorticotropic hormone (ACTH) analogue, has demonstrated potential benefit in small randomized trials. Proposed mechanisms include stimulation of CSF production and stabilization of meningeal membranes [21].

Another strategy in obstetric anesthesia involves leaving an intrathecal catheter in place following UDP. Maintaining the catheter for approximately 24 hours may reduce PDPH incidence by promoting inflammatory sealing of the dural defect and decreasing continued CSF leakage [22].

Prophylactic EBP has also been studied; however, randomized trials have not demonstrated consistent benefit, and routine use remains controversial [23].

Therapeutic management

Conservative Therapy

**Figure 2:**
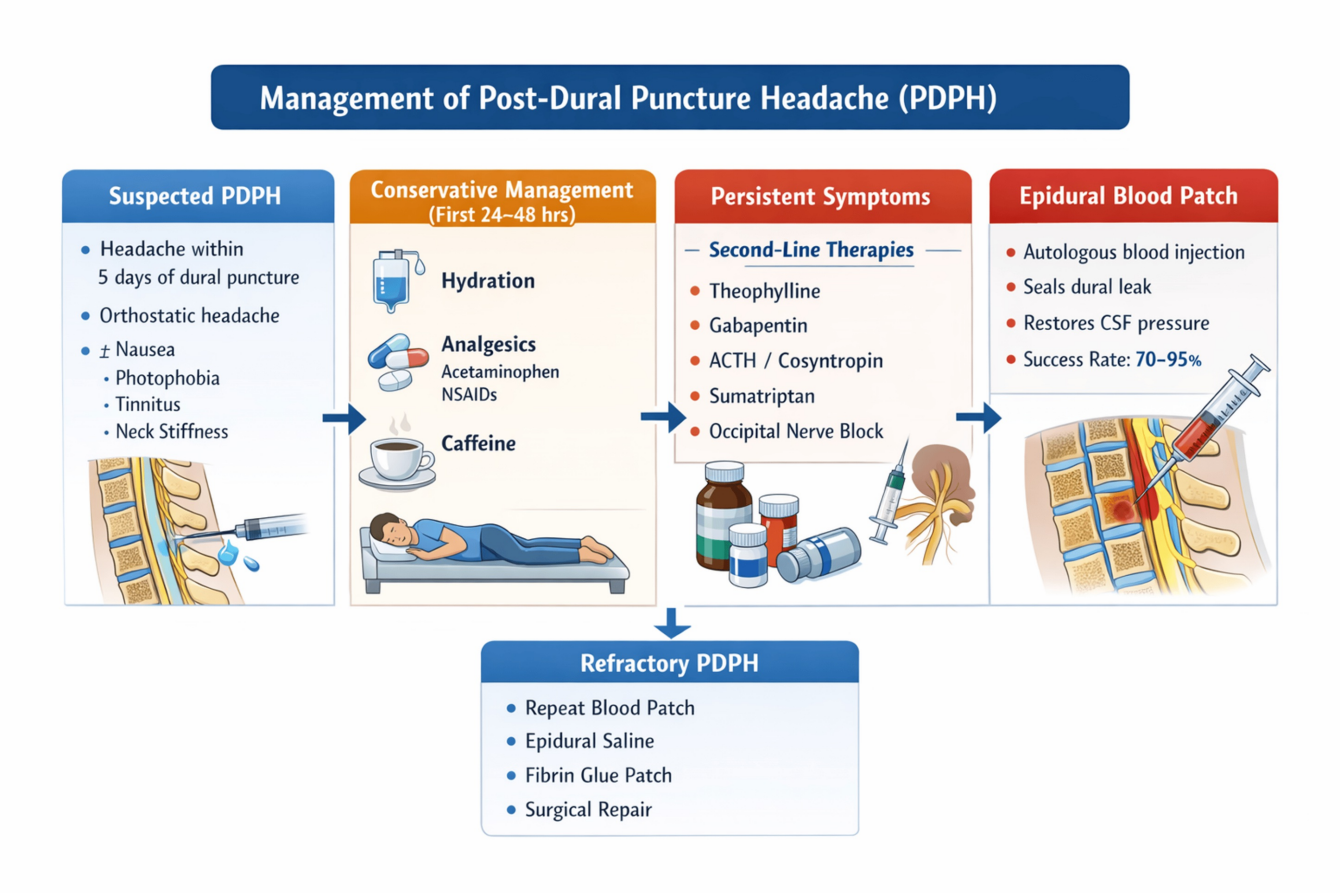
Clinical algorithm for management of post-dural puncture headache (PDPH) Initial treatment involves conservative measures, including hydration, oral analgesics, caffeine, and limited bed rest. Patients with persistent or moderate-to-severe symptoms may benefit from pharmacologic therapies such as theophylline, gabapentin, cosyntropin, or nerve blocks. The epidural blood patch remains the definitive treatment, sealing the dural defect and restoring CSF pressure with high success rates. Refractory cases may require repeat blood patch or alternative interventional approaches. Image credit: The authors. Figure created with BioRender.com.

Initial management of PDPH (Figure 2) typically involves conservative therapies aimed at symptomatic relief while spontaneous closure of the dural defect occurs.

Recommended first-line interventions include adequate hydration, oral analgesics, such as acetaminophen or nonsteroidal anti-inflammatory drugs (NSAIDs), and caffeine administration [20].

Caffeine may be administered orally at doses of 300-500 mg once or twice daily, or intravenously as 500 mg of caffeine sodium benzoate, which may be repeated after 24 hours if necessary. Combination preparations containing caffeine and analgesics (e.g., butalbital-acetaminophen-caffeine (Fioricet)), and acetaminophen-aspirin-caffeine (Excedrin) may also be utilized as adjunctive therapy for symptom control [20].

Nonpharmacologic adjuncts, such as abdominal binders, have been proposed to increase intra-abdominal pressure and reduce CSF leakage by elevating epidural venous pressure. However, supporting evidence is sparse, and these interventions are generally considered optional supportive measures rather than primary therapy.

Although these measures may provide partial relief, they rarely result in the complete resolution of symptoms in patients with persistent CSF leakage.

Additional Pharmacologic Therapies

Several pharmacologic agents have been investigated as alternatives or adjuncts to EBP.

Gabapentin and pregabalin have demonstrated modest analgesic benefit in small randomized trials, possibly through modulation of central sensitization pathways [24].

Theophylline, a methylxanthine derivative similar to caffeine, has also shown efficacy in reducing headache intensity by promoting cerebral vasoconstriction and increasing CSF production [25].

Sumatriptan, a serotonin receptor agonist, has been explored based on its effects on cranial vasculature, although evidence remains inconsistent [26].

More recently, cosyntropin (ACTH analogue) has gained attention due to its potential ability to increase CSF production and stimulate endogenous corticosteroid release [21].

Epidural Blood Patch

EBP remains the gold-standard treatment for moderate to severe PDPH [27]. The procedure involves the injection of autologous venous blood into the epidural space near the site of dural puncture. The injected blood forms a clot that seals the dural defect and reduces ongoing CSF leakage. Additionally, the injected volume increases epidural pressure, transiently restoring intracranial CSF pressure and alleviating symptoms.

Clinical success rates following the first EBP range from approximately 70-90% [27]. Patients who experience partial improvement may benefit from a second procedure. Studies have suggested that injection of approximately 20 mL of autologous blood provides optimal therapeutic efficacy, although smaller volumes may be used depending on patient tolerance [28].

Predictors of blood patch success: Although the EBP remains widely performed, several factors influence procedural success. Observational studies suggest that delayed administration (>24 hours after dural puncture) may be associated with higher success rates due to partial closure of the dural defect and stabilization of CSF dynamics [29]. Conversely, early blood patching may be associated with increased failure rates and the need for repeat procedures. Predictors of unsuccessful blood patch include large dural defects, multiple puncture attempts, connective tissue disorders, and persistent high-flow CSF leaks. Recent imaging studies utilizing MRI and CT myelography have been proposed to localize persistent leaks in refractory cases [30].

Complications are uncommon but may include transient back pain, radicular symptoms, infection, or epidural hematoma.

Alternative therapies

Alternative therapeutic approaches have been explored for patients who cannot undergo an EBP or who experience incomplete symptom relief from conservative measures.

Greater Occipital Nerve Blockade (GONB)

GONB is a minimally invasive option for managing PDPH [31]. The greater occipital nerve, arising from the dorsal ramus of C2, provides sensory innervation to the posterior scalp and contributes to headache pain pathways. The block involves injection of local anesthetic, with or without corticosteroid, near the nerve as it emerges along the superior nuchal line. GONB is thought to relieve PDPH by interrupting nociceptive input and modulating the trigeminocervical complex. Small studies and case reports suggest rapid symptom relief in some patients, although evidence remains limited compared with EBP [31]. It may serve as a useful adjunct or temporizing treatment within a multimodal approach to PDPH management.

Intranasal Sphenopalatine Ganglion Block (SPGB)

Intranasal SPGB is a minimally invasive option for the management of post-dural puncture [32]. The sphenopalatine ganglion, located in the pterygopalatine fossa posterior to the middle nasal turbinate, contains parasympathetic fibers involved in cerebral vasodilation, a mechanism implicated in PDPH following CSF leakage and intracranial hypotension. The technique involves transnasal application of local anesthetic (commonly lidocaine or ropivacaine) using cotton-tipped applicators or specialized devices to block parasympathetic activity. Small studies and case series have reported rapid symptom relief with minimal adverse effects such as transient nasal discomfort or epistaxis, making intranasal SPGB a useful adjunct in the stepwise management of PDPH [32].

Although these interventions do not address the underlying CSF leak, they may serve as useful adjuncts for symptom management in selected patients.

Emerging and investigational therapies

Novel therapeutic strategies continue to be explored for patients with refractory PDPH. These approaches include fibrin glue, epidural injections, epidural saline infusion, and targeted EBP placement guided by advanced imaging modalities such as CT myelography [30]. Advances in imaging techniques, particularly dynamic CT myelography and digital subtraction myelography, have significantly improved the ability to localize CSF leaks in patients with persistent or atypical symptoms, thereby enabling more precise and individualized interventions [30].

Neostigmine and Atropine

Intravenous administration of neostigmine in combination with atropine has been studied as a potential therapeutic option for PDPH. Proposed mechanisms include augmentation of parasympathetic tone, modulation of cerebral vascular tone, and stimulation of CSF production through cholinergic pathways. Small randomized trials [33] have demonstrated reductions in headache severity and decreased analgesic requirements; however, current evidence is limited by small sample sizes and heterogeneity in study design. Larger, well-designed randomized controlled trials are necessary before widespread clinical adoption can be recommended.

In addition, growing interest exists in identifying biomarkers associated with neuroinflammation and connective tissue integrity that may predict susceptibility to PDPH and response to treatment. Advances in this area may allow for risk stratification, earlier intervention, and more personalized therapeutic strategies in the future.

Epidural Saline/Dextran or Dextrose

Epidural administration of preservative-free normal saline has been proposed as an alternative or temporizing therapy for PDPH [34]. Injection of approximately 10-30 mL of saline into the epidural space may transiently increase epidural pressure and partially restore CSF pressure, leading to temporary improvement in orthostatic headache symptoms. However, symptom relief is typically short-lived due to rapid redistribution and resorption of saline. Epidural administration of dextran or dextrose-containing solutions has also been investigated with the aim of prolonging epidural volume expansion and enhancing the durability of symptom relief. Nevertheless, available evidence supporting the routine use of these agents remains limited when compared with the established efficacy of EBP [34].

Delayed and rare sequelae

Persistent intracranial hypotension may predispose to subdural hematoma due to bridging vein traction [35]. Cerebral venous thrombosis may occur secondary to venous stasis [12].

Prospective studies have demonstrated that patients experiencing severe PDPH may have an increased risk of chronic headache syndromes and persistent back pain months to years after the initial dural puncture. Amorim et al. reported that PDPH was associated with a significantly higher risk of new-onset chronic headache compared with matched controls [35].

In obstetric populations, Webb et al. highlighted prolonged postpartum morbidity following UDP [11]. UDP has been linked to increased incidence of postpartum depression, prolonged recovery, and reduced maternal functional status, highlighting the broader psychosocial impact of this complication. Although the absolute incidence of serious complications is low, clinicians must maintain vigilance for neurologic deterioration.

Critical synthesis and future directions

Despite decades of investigation into PDPH, several fundamental questions remain unresolved. The optimal timing of EBP continues to lack high-quality randomized controlled data to guide decision-making, particularly in differentiating early intervention from expectant management strategies.

Similarly, reliable predictive models to distinguish patients likely to experience spontaneous resolution from those with persistent CSF leak have not yet been developed. The role of inflammatory amplification in symptom severity remains poorly characterized, and potential biomarkers that may correlate with headache intensity, duration, or treatment response remain largely unexplored.

Furthermore, long-term outcomes - including neurocognitive sequelae and chronic headache phenotypes - require prospective longitudinal investigation to better understand the broader implications of PDPH. In obstetric populations, standardized management pathways for UDP require further refinement to balance efficacy, safety, and maternal-infant considerations.

Future research should move toward an integrated framework incorporating advanced imaging modalities, spinal compliance modeling, and biomarker profiling to facilitate risk stratification and ultimately enable personalized management strategies.

## Conclusions

PDPH remains a clinically significant complication of neuraxial procedures despite advances in needle design and procedural technique that have substantially reduced its incidence. Current evidence supports a multifactorial pathophysiologic model involving CSF volume depletion, compensatory intracranial vascular changes, neural traction, and potential neuroinflammatory contributions, which collectively produce the characteristic orthostatic headache phenotype. Procedural factors, particularly needle gauge and tip configuration, represent the most important modifiable determinants of risk, with atraumatic small-gauge needles significantly reducing PDPH incidence compared with larger cutting needles or UDP. Management strategies range from conservative therapies to EBP, which remains the most effective treatment for moderate to severe cases, while emerging therapies and targeted interventions may expand future treatment options. Continued research integrating advanced imaging, biomarker identification, and improved risk stratification may facilitate more individualized prevention and management strategies, ultimately improving patient outcomes.
